# Association between dietary index for gut microbiota and osteoarthritis in the US population: the mediating role of systemic immune-inflammation index

**DOI:** 10.3389/fnut.2025.1543674

**Published:** 2025-04-28

**Authors:** Jiulong Song, Jian Fu

**Affiliations:** College of Physical Education, Yangzhou University, Yangzhou, Jiangsu, China

**Keywords:** osteoarthritis, dietary index for gut microbiota, systemic immune-inflammation index, US population, mediation effect

## Abstract

**Objective:**

Osteoarthritis (OA) is one of the most prevalent chronic conditions among the elderly. The dietary index for gut microbiota (DI-GM) is a novel proposed indicator reflecting gut microbiome diversity. However, the role of DI-GM in OA remains unclear. This study thus aims to explore the association between DI-GM and the risk of OA and analyze the mediating roles of systemic immune-inflammation index (SII).

**Methods:**

We utilized data from the National Health and Nutrition Examination Survey (NHANES) spanning 2007-2018. OA was assessed through self-reported questionnaires, and dietary recall data were used to calculate the DI-GM. Univariate and weighted multivariate logistic regression analyses were employed to evaluate the association between DI-GM and OA, the weighted linear regression analyses were employed to investigate the association of DI-GM with SII, while restricted cubic splines (RCS) curves were used to assess the non-linear relationship between these variables. Subgroup analyses were subsequently conducted to validate the robustness of the findings. Mediation analysis evaluated the role of SII.

**Results:**

This study included 15,875 participants, revealing a significant inverse association between the DI-GM and OA risk (*p* < 0.001), higher DI-GM demonstrated a substantially reduced OA risk (adjusted model OR: 0.83; 95% CI: 0.79–0.86) and were negatively associated with the SII [β (95% CI): –9.2 (–13.0, –2.0)]. The RCS curve indicated a non-linear relationship between DI-GM and OA risk. Subgroup analysis showed that various demographic and clinical factors did not significantly alter the association between DI-GM and OA risk (interaction *p*-value > 0.05). The mediating effect of SII accounted for 12.69% of association between DI-GM and OA.

**Conclusion:**

This study found a significant negatively association between DI-GM and OA prevalence in the US population. Mediation analyses demonstrated a significant mediating effect of SII.

## 1 Introduction

Osteoarthritis (OA) is a common chronic degenerative joint disease characterized by the deterioration of articular cartilage and structural changes in the joint (1). It most frequently affects large joints such as the knees, hips, and hands, leading to symptoms including pain, stiffness, and impaired function (2). Epidemiological data indicate that approximately 18% of women and 10% of men worldwide develop OA after the age of 60 (3). In the United States, more than 32 million adults are affected by OA, and projections suggest that this number will rise to 67 million by 2030 (4). With the ongoing global trend of aging, the prevalence and disability rates associated with OA continue to increase, placing a growing burden on public health systems and socioeconomic development (5, 6).

The gut microbiota is a diverse and complex community of microorganisms inhabiting the gastrointestinal tract, consisting of approximately 35,000 bacterial species that form the gut microbiome (7). It plays a critical role in nutrient absorption, maintaining metabolic balance, regulating the immune system, and reducing systemic inflammation (8). Recently, Kase et al. developed the Dietary Gut Microbiome Index (DI-GM) (9), a dietary measure designed to support a healthy gut microbiota. This index was created by analyzing the relationship between various foods or food groups and the composition of the adult gut microbiota. Based on its correlation with indirect biomarkers of gut microbiota diversity, the DI-GM effectively identifies dietary patterns that promote or hinder gut microbiota health, providing a standardized tool that can measure diet quality associated with maintain healthy gut microbiota.

The systemic immune-inflammation index (SII) is a composite biomarker derived from peripheral blood cell counts, providing a comprehensive reflection of the body’s inflammatory and immune status (10). Recent studies have indicated that SII is abnormal elevated in various chronic inflammatory conditions and is strongly linked to disease severity and prognosis (11). OA as a prevalent chronic degenerative disorder, involves both local and systemic inflammatory responses in its pathogenesis. Emerging evidence suggests that an elevated SII is positively correlated with pain severity and joint dysfunction in patients with OA (12). Additionally, higher SII level have been significantly associated with the radiographic severity of OA (13), indicating that SII could serve as a potential biomarker for assessing disease activity and progression.

There is growing interest in modulating the gut microbiota through dietary approaches. An increasing body of research suggests that the gut microbiota may influence the function of host tissue and organs, including inflammatory status (14), the gut microenvironment (15), and joint integrity (16). Recent study indicate that gut microbiota and probiotics can reduce cartilage degeneration during OA flare-ups (17). Meanwhile, increasing attention has been directed toward the role of dietary components in modulating immune responses and inflammatory processes within the body. Previous study has shown that gut microbiota dysbiosis might exacerbate intra-articular inflammatory responses in OA patients by impairing intestinal barrier integrity and promoting the release of pro-inflammatory cytokines (18). Therefore, exploring the association between DI-GM and OA, as well as the mediating role of SII, holds significant scientific value.

Although previous studies have focused on the direct modulation of gut microbiota, few studies have indirectly assessed how gut microbiota modulate SII to reduce the risk of OA. We conducted a cross-sectional study using data from the National Health and Nutrition Examination Survey (NHANES) to explore the potential association between DI-GM and OA. Additionally, we assessed the mediating role of the SII in this relationship. Our study provides deeper insights into the complex interplay among dietary intake, gut microbiota, and SII, offering novel scientific evidence to inform the prevention and intervention strategies for OA.

## 2 Materials and methods

### 2.1 Study design and population

This study is based on data from the National Health and Nutrition Examination Survey (NHANES), a comprehensive, nationally representative cross-sectional survey conducted by the National Center for Health Statistics (NCHS). The survey employs a multi-stage probability sampling design, with data released every 2 years. We included 6 distinct NHANES data cycles from 2007 to 2018, focusing on adult participants aged 20-80 years. Participants provided information on OA diagnoses, DI-GM, and related variables. The NCHS Ethics Review Board reviewed and approved this study, participants provided written informed consent, and the research utilized anonymized publicly available NHANES data.

**FIGURE 1:**
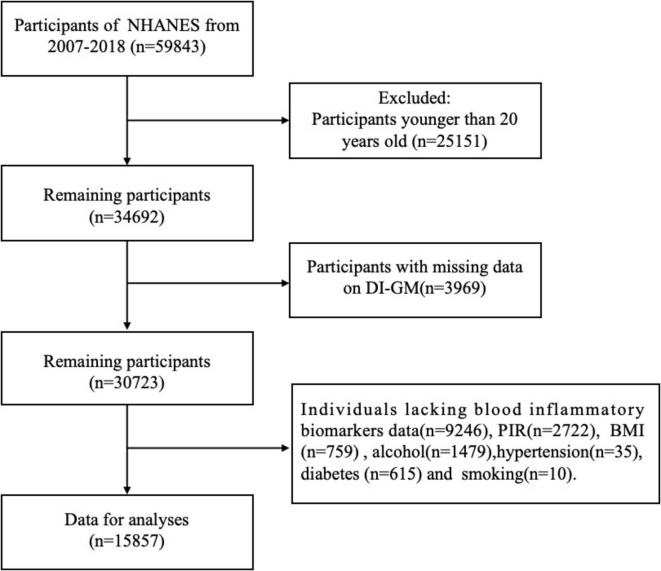
Flowchart of participant inclusion and exclusion criteria.

A total of 59,843 individuals participated in this study. Exclusions were made for individuals under the age of 20 years (25,151), and individuals lacking DI-GM data (*n* = 3,969). Additional exclusions included participants with missing information on blood inflammatory biomarkers (*n* = 9,246), poverty income ratio (PIR) (*n* = 2,722), body mass index (BMI) (*n* = 759), alcohol consumption (*n* = 1,479), hypertension (*n* = 35), diabetes (*n* = 615), and smoking status (*n* = 10). Ultimately, 15,857 participants were included in the final analysis (Figure 1).

### 2.2 Definitions of dietary index for gut microbiota

Participants’ consumption of the Diversity Index of Gut Microbiota (DI-GM) was assessed through dietary recall interviews using data from the NHANES 2007-2018. Dietary data were collected via two interviewer-administered 24-h dietary recall components, employing the automated multiple-pass method developed by the United States Department of Agriculture (USDA). The DI-GM index used in this study was developed and validated by Kase et al. (9) based on the NHANES database. Given that our study data were also derived from NHANES, the validity and applicability of this index are more strongly support in the present study. The DI-GM score is based on 14 foods and nutrients. Beneficial components include fermented dairy products, chickpeas, soybeans, whole grains, dietary fiber, cranberries, avocados, broccoli, coffee, and green tea. Adverse components include refined grains, red meat, processed meat, and high-fat diets (≥ 40% of energy from fat). For each beneficial component, a score of 1 is assigned when consumption exceeds the sex-specific median, and a score of 0 is assigned when intake falls below this threshold. The scores for each component are summed to yield a total DI-GM score ranging from 0 to 14, with beneficial food contributing 0-10 points and unfavorable foods 0-4 points. Participants were divided into four groups based on quartiles of total scores: 0-3, 4, 5, and ≥ 6 (19). The components and scoring criteria of DI-GM are detailed in Supplementary Table 1.

### 2.3 Diagnosis of OA

OA diagnosis data were assessed using the Medical Conditions questionnaire from the NHANES database. This questionnaire includes the question, “Has a doctor or other health professional ever told you that you have arthritis?” with response options of “yes” or “no.” Participants who answered, “yes” proceeded to the subsequent set of questions, which asked, “What type of arthritis was it?” Individuals who selected the OA option were included in the study (20).

### 2.4 Assessment of SII

The Systemic Immune-Inflammation Index (SII) was measured to assess immune-related inflammation in participants, providing an objective reflection of changes in the body’s inflammatory levels (21). This index incorporates platelet count, lymphocyte count, and neutrophil count. Blood samples from all participants were collected at the mobile examination center following NHANES protocols, and quantitative assessments of blood components were performed using the Beckman Coulter method. The SII was calculated by multiplying the platelet count by the neutrophil count and then dividing by the lymphocyte count, as reported in previous research (22).

### 2.5 Covariates

Drawing from prior research (23, 24), we compiled a comprehensive set of covariates known to influence OA as potential confounders. These included demographic factors such as age (20-39, 40-59, ≥ 60), gender (male or female), and race (Non-Hispanic White, Non-Hispanic Black, Mexican American, Other Race and Other Hispanic); education level (<9th grade, 9-11th grade, High school graduate, Some college or AA degree and College graduated or above) and poverty-to-income ratio (PIR) (<1.30, 1.30-3.49, ≥ 3.50); body mass index (BMI) (<25, 25-30, > 30 kg/m^2^). We also included self-reported chronic conditions, including diabetes and hypertension. Smoking status was categorized based on whether participants had smoked at least 100 cigarettes in their lifetime and whether they currently smoke. Alcohol consumption was categorized as consuming at least 12 alcoholic beverages per year, enabling us to assess their potential influence on the study outcomes.

### 2.6 Statistical analysis

In our statistical analysis, we applied weights to the corresponding samples across different cycles of the NHANES data. Continuous variables are expressed as means and standard errors (SE), while categorical variables are represented by frequencies (n) and percentages (%). The chi-square test was used to assess differences in categorical variables, while differences in continuous variables were evaluated using the *t*-test (for variables with a normal distribution) or the Mann–Whitney test (for variables with a skewed distribution). Univariable and weighted multivariable logistic regression analyses were performed, with results presented as odds ratios (OR) and 95% confidence intervals (CI). Univariable analysis was conducted to examine the association between each variable and OA. To explore the relationship between DI-GM and OA, we applied weighted multivariable logistic regression models. Additionally, multivariable weighted linear regression was conducted to evaluate associations between the DI-GM and the SII. Model I was unadjusted for covariates; Model II adjusted for demographic factors including age, sex, race, educational level, and poverty–income ratio, and Model III further adjusted for variables such as BMI, smoking status, alcohol consumption, hypertension, diabetes, and SII.

We utilized restricted cubic spline (RCS) regression to evaluate potential non-linear relationships between DI-GM and OA. Furthermore, we conducted an in-depth investigation into the potential mediating role of the SII in the association between DI-GM and OA. Mediation analysis was performed using the Sobel test and the bootstrap method, with 1,000 resampling iterations to compute the 95% CI for the mediation effect. The mediation effect was quantified as the proportion mediated. Sensitivity analyses included subgroup analysis and propensity score matching (PSM). We performed subgroup analyses based on age, sex, race, educational level, PIR, BMI, smoking status, alcohol use, diabetes, hypertension, and the SII to explore potential interactions between these covariates and the DI-GM and OA association. Based on previous evidence indicating that these factors may influence dietary habits, gut microbiota composition, inflammation levels, and OA risk (25, 26). Interaction tests were also conducted to evaluate heterogeneity across subgroups. The PSM was performed with the OA population serving as the reference group, using a 1:1 matching ratio to further eliminate bias and control for potential confounding baseline variables between groups. Subsequently, logistic regression analyses were conducted on the matched samples. Detailed information regarding the PSM methodology is provided in the Supplementary material. All data extraction and analyses were performed using R software (version 4.4.1),^1^ with statistical significance defined as *p* < 0.05.

## 3 Results

### 3.1 Baseline characteristics of the study participants

In this study, a total of 15,875 participants were stratified according to OA status using NHANES data from 2007 to 2018. Of these, 5,032 individuals had OA, while 10,825 were without OA. OA participants were significantly older than non-OA participants, with 47.5% being ≥ 60 years of age. The proportion of females was higher in the OA group (58%) compared to the non-OA group. Among racial groups, non-Hispanic White participants had the highest OA prevalence (76%), followed by non-Hispanic Black participants (9.7%, *p* < 0.001). Educational attainments also differed significantly between groups, with a smaller proportion of OA patients being college graduates (25%) compared to the non-OA (33%), and a larger proportion of OA participants having a poverty-income ratio (PIR) below 1.30 (*p* < 0.001). Additionally, OA patients had higher SII values (*p* < 0.001) and lower DI-GM scores, along with a higher prevalence of comorbidities such as hypertension and diabetes (*p* < 0.001), OA participants also reported higher alcohol consumption (*p* = 0.006) and slightly lower smoking rates (*p* < 0.001). Detailed baseline characteristics of all participants grouped by OA status are presented in Table 1.

**TABLE 1:** Baseline characteristics of the study participants grouped by osteoarthritis status.

Characteristic	Osteoarthritis			
	**Overall, *N*^1^ = 15,875 (100%)^2^**	**Non-OA *N* = 10,825 (71%)^2^**	**OA *N* = 5,032 (29%)^2^**	***P*-value^3^**
**Age (years), n (%)**				**<0.001**
20-39	5,361 (36.7%)	4,678 (45.9%)	683 (14.5%)	
40-59	5,131 (37.1%)	3,614 (36.7%)	1,517 (38.0%)	
≥ 60	5,365 (26.2%)	2,533 (17.4%)	2,832 (47.5%)	
**Sex, n (%)**				**<0.001**
Male	7,869 (49%)	5,676 (52%)	2,193 (42%)	
Female	7,988 (51%)	5,149 (48%)	2,839 (58%)	
**Race, n (%)**				**<0.001**
Non-Hispanic White	7,019 (69%)	4,392 (67%)	2,627 (76%)	
Non-Hispanic Black	3,331 (10.0%)	2,228 (10%)	1,103 (9.7%)	
Mexican American	2,090 (7.9%)	1,559 (9.2%)	531 (5.0%)	
Other Race	1,918 (7.3%)	1,591 (8.2%)	327 (5.2%)	
Other Hispanic	1,499 (5.5%)	1,055 (6.0%)	444 (4.1%)	
**Education, n (%)**				**<0.001**
<9th grade	1,307 (4.2%)	750 (3.5%)	557 (5.7%)	
9-11th grade	2,158 (10.0%)	1,397 (9.4%)	761 (11%)	
High school graduate	3,661 (23%)	2,410 (23%)	1,251 (25%)	
Some college or AA degree	4,846 (32%)	3,319 (31%)	1,527 (33%)	
College graduate or above	3,885 (31%)	2,949 (33%)	936 (25%)	
**PIR, n (%)**				**0.021**
< 1.30	4,910 (21.15%)	3,230 (20.72%)	1,680 (22.18%)	
1.30-3.49	5,876 (34.70%)	3,940 (34.04%)	1,936 (36.28%)	
≥ 3.50	5,071 (44.16%)	3,655 (45.24%)	1,416 (41.54%)	
**BMI (Kg/m^2^)**				**<0.001**
<25	4,612 (29%)	3,502 (33%)	1,110 (22%)	
25-30	5,956 (37%)	3,652 (33%)	2,304 (46%)	
> 30	5,289 (34%)	3,671 (34%)	1,618 (32%)	
**Hypertension, n (%)**				**<0.001**
Yes	5,689 (32%)	2,950 (24%)	2,739 (50%)	
No	10,168 (68%)	7,875 (76%)	2,293 (50%)	
**Diabetes, n (%)**				**<0.001**
Yes	2,118 (9.9%)	1,043 (7.2%)	1,075 (17%)	
No	13,739 (90%)	9,782 (93%)	3,957 (83%)	
**Smoking, n (%)**				**<0.001**
Nonsmoker	8,750 (56%)	6,418 (59%)	2,332 (47%)	
Smoker	7,107 (44%)	4,407 (41%)	2,700 (53%)	
**Alcohol, n (%)**				**0.006**
Nondrinker	3,598 (18%)	2,351 (17%)	1,247 (20%)	
Drinker	12,259 (82%)	8,474 (83%)	3,785 (80%)	
**SII (mean** ± **SE)**	538 ± (3.13)	525 ± (2.96)	570 ± (3.49)	**<0.001**
**DI_GM (mean** ± **SE)**	5.06 ± (0.02)	5.18 ± (0.06)	4.80 ± (0.04)	**<0.001**
**DI_GM n (%)**				**<0.001**
0-3	3,060 (18%)	1,557 (14%)	1,503 (27%)	
4	3,429 (21%)	2,457 (21%)	972 (19%)	
5	3,685 (23%)	2,704 (24%)	981 (19%)	
≥ 6	5,683 (39%)	4,107 (40%)	1,576 (35%)	

^1^N not missing

^2^Median (IQR) for continuous; n (%) for categorical

^3^Pearson’s X^^2^: Rao & Scott adjustment Design-based Kruskal Wallis test. Categorical variables were compared using Pearson’s chi-square tests between OA and non-OA groups. OA, Osteoarthritis; PIR, poverty–income ratio; BMI, body mass index; SII, systemic inflammation index; SE, standard errors; DI-GM, dietary index for gut microbiota. The DI-GM ranges from 0 to 14 and grouped according to 0–3, 4, 5, and ≥ 6. Bold values indicate primary indicators and statistically significant results.

### 3.2 Univariate analysis

Table 2 presents the result of univariate analyses for each variable about OA. Our findings show that, compared to individuals aged 20–39 years, those aged 40–59 had 2.49 times higher odds of developing OA, while individuals aged ≥ 60 exhibited a 2.81-fold increase in odds. Females were 1.65 times more likely to develop OA than males. Mexican American participants (OR: 1.89; *p* < 0.001) and individuals of other racial backgrounds (OR: 1.80; *p* < 0.001) had higher OA prevalence compared to non-Hispanic White participants. Higher educational attainment was negatively associated with OA risk (OR: 0.64; *p* < 0.001). Similarly, individuals with a PIR ≥ 3.50 (OR: 0.63; *p* < 0.001) had a lower risk of OA compared to those with a PIR < 1.30. Participants with a BMI > 30 (OR: 1.72; *p* < 0.001) had significantly higher odds of OA compared to those with a BMI < 25. OA risk was also higher in individuals with hypertension or diabetes than in those without these conditions. Additionally, smokers and alcohol consumers were more likely to develop OA compared to non-smokers and non-drinkers. Finally, a positive correlation was observed between SII values and OA prevalence.

**TABLE 2:** Univariate analysis of factors associated with osteoarthritis status.

Characteristic	Estimate	SE	*t*-value	OR	95% CI	*P*-value
**Age**						
20-39	1.0 (ref)	1.0 (ref)	1.0 (ref)	1.0 (ref)	1.0 (ref)	1.0 (ref)
40-59	0.911	0.06	14.78	2.49	2.20, 2.81	**<0.001**
≥ 60	1.03	0.06	15.35	2.81	2.46, 3.21	**<0.001**
**Sex, n (%)**						
Male	1.0 (ref)	1.0 (ref)	1.0 (ref)	1.0 (ref)	1.0 (ref)	1.0 (ref)
Female	0.49	0.05	9.85	1.65	1.49, 1.82	**<0.001**
**Race, n (%)**						
Non-Hispanic White	1.0 (ref)	1.0 (ref)	1.0 (ref)	1.0 (ref)	1.0 (ref)	1.0 (ref)
Non-Hispanic Black	0.21	0.10	1.99	1.26	0.98, 1.63	0.049
Mexican American	0.63	0.07	8.71	1.89	1.63, 2.18	**<0.001**
Other Race	0.58	0.07	7.41	1.80	1.54, 2.11	**<0.001**
Other Hispanic	0.24	0.12	2.03	1.28	1.01, 1.63	0.045
**Education, n (%)**						
<9th grade	1.0 (ref)	1.0 (ref)	1.0 (ref)	1.0 (ref)	1.0 (ref)	1.0 (ref)
9-11th grade	–0.13	0.09	–1.43	0.74	0.61, 0.91	0.006
High school graduate	–0.19	0.08	–2.17	0.68	0.55, 0.84	**<0.001**
Some college or AA degree	–0.20	0.08	–2.30	0.66	0.52, 0.84	0.001
College graduate or above	–0.62	0.09	–6.63	0.64	0.54, 0.75	**<0.001**
**PIR, n (%)**						
<1.30	1.0 (ref)	1.0 (ref)	1.0 (ref)	1.0 (ref)	1.0 (ref)	1.0 (ref)
1.30–3.49	–0.16	0.05	–3.06	0.85	0.77, 0.95	0.003
≥3.50	–0.46	0.06	–7.24	0.63	0.55, 0.71	**<0.001**
**BMI (Kg/m^2^)**						
<25	1.0 (ref)	1.0 (ref)	1.0 (ref)	1.0 (ref)	1.0 (ref)	1.0 (ref)
25–30	0.15	0.06	2.43	1.16	1.03, 1.31	0.017
>30	0.54	0.05	9.63	1.72	1.54, 1.93	**<0.001**
**Hypertension, n (%)**						
No	1.0 (ref)	1.0 (ref)	1.0 (ref)	1.0 (ref)	1.0 (ref)	1.0 (ref)
Yes	0.79	0.04	18.23	2.22	2.03, 2.42	**<0.001**
**Diabetes, n (%)**						
No	1.0 (ref)	1.0 (ref)	1.0 (ref)	1.0 (ref)	1.0 (ref)	1.0 (ref)
Yes	0.51	0.06	8.14	1.68	1.48, 1.91	**<0.001**
**Smoking, n (%)**						
Nonsmoker	1.0 (ref)	1.0 (ref)	1.0 (ref)	1.0 (ref)	1.0 (ref)	1.0 (ref)
Smoker	0.31	0.04	6.75	1.37	1.25, 1.51	**<0.001**
**Alcohol, n (%)**						
Nondrinker	1.0 (ref)	1.0 (ref)	1.0 (ref)	1.0 (ref)	1.0 (ref)	1.0 (ref)
Drinker	0.71	0.05	9.31	1.84	1.75, 1.94	0.002
**SII**	0.72	0.04	12.88	1.63	1.54, 1.87	0.003

OR, Odds Ratio; CI, Confidence Interval; ref, reference; PIR, poverty–income ratio; BMI, body mass index; SII, systemic inflammation index; SE, standard errors; DI-GM, dietary index for gut microbiota. Bold values indicate primary indicators and statistically significant results.

### 3.3 Association between DI-GM and OA

**FIGURE 2:**
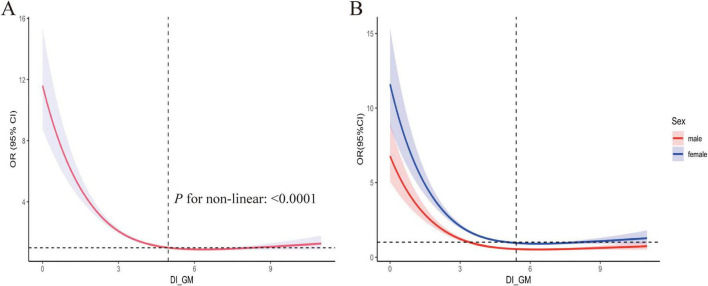
Restricted cubic spline fitting for the association between DI_GM and OA. **(A)** Adjustments were made for Age, Sex, Race, Educational, poverty-income ratio (PIR), body mass index (BMI), Diabetes, Hypertension, Smoking and Alcohol status, as well as the systemic inflammation index (SII). **(B)** Stratified analysis was conducted by sex, with the red curve representing males and the blue curve representing females. DI-GM, dietary index for gut microbiota, OA, osteoarthritis.

Table 3 presents the results of weighted multivariate logistic regression analyses examining the relationship between DI-GM and OA risk. Three models were constructed, each adjusted for potential confounders, revealing an inverse association between DI-GM and OA risk. The OR with 95% CI for each model were as follows: Model I 0.88 (0.85, 0.91), Model II 0.82 (0.78, 0.85), and Model III 0.83 (0.79–0.86). To assess the robustness of these findings, DI-GM was stratified into quartiles. In all three models, a persistent inverse relationship between DI-GM and OA was observed. In the fully adjusted Model III, the DI-GM ≥ 6 group was 0.55 (0.46-0.65), indicating that higher DI-GM values were associated with a significant decrease in OA prevalence. Additionally, RCS curves were used to explore the potential non-linear relationship between DI-GM and OA. As shown in Figure 2A, the RCS curve indicates a non-linear association between DI-GM and OA. Further analysis revealed gender-based differences in the effect of OA. Both males and females showed similar non-linear relationships between DI-GM and OA risk (Figure 2B).

**TABLE 3:** Association between DI-GM and osteoarthritis analyzed using logistic regression.

	Model I		Model II		Model III	
	OR (95% CI)	*P*-value	OR (95% CI)	*P*-value	OR (95% CI)	*P*-value
**DI-GM**	0.88 (0.85, 0.91)	**<0.001**	0.82 (0.78, 0.85)	**<0.001**	0.83 (0.79, 0.86)	**<0.001**
**DI-GM group**						
0–3	Ref	–	Ref	–	Ref	–
4	0.70 (0.62, 0.79)	**<0.001**	0.63 (0.55, 0.73)	**<0.001**	0.67 (0.54, 0.74)	**<0.001**
5	0.68 (0.59, 0.79)	**<0.001**	0.58 (0.49, 0.69)	**<0.001**	0.60 (0.50, 0.72)	**<0.001**
≥6	0.68 (0.60, 0.78)	**<0.001**	0.52 (0.44, 0.61)	**<0.001**	0.55 (0.46,0.65)	**<0.001**
***P* for trend**	**0.002**		**<0.001**		**<0.001**	

OR, Odds Ratio; CI, Confidence Interval; DI-GM, dietary index for gut microbiota. The DI-GM ranges from 0–14 and grouped according to 0–3, 4, 5, and ≥ 6. Model I was unadjusted for covariates; Model II was adjusted for age, sex, race, educational level and poverty–income ratio; Model III was adjusted for age, sex, race, educational level and poverty–income ratio, BMI (body mass index), smoking status, alcohol consumption, hypertension, diabetes, and systemic inflammation index (SII). Bold values indicate primary indicators and statistically significant results.

### 3.4 Association between DI-GM and SII

The association between DI-GM and SII was examined using weighted linear regression models. As shown in Table 4, a negative correlation between higher DI-GM and SII was observed across all three models (Model I, Model II, and Model III). Specifically, Model I yielded a β (95% CI) of –8.8 (–12.0, –1.5), *p* = 0.004; Model II, β (95% CI): –9.7 (–12.8, –1.7), *p* < 0.001; and Model III, β (95% CI): –9.2 (–13.0, –2.0), *p* = 0.002. These results indicated that a one-point increase in the DI-GM score is associated with a 9.2% reduction in the SII index.

**TABLE 4:** The association between DI-GM and systemic immune inflammation index.

	Model I		Model II		Model III	
	β (95% CI)	*P-*value	β (95% CI)	*P*-value	β (95% CI)	*P*-value
DI-GM	–6.4 (–10.0, –2.4)	**0.002**	–8.5 (–13.0, –4.3)	**<0.001**	–7.3 (–12.0, –3.0)	**0.001**
**DI-GM group**						
0–3	Ref	–	Ref	–	Ref	–
4	–4.5 (–7.8, 2.3)	0.60	–5.3 (–9.7.0, 2.0)	0.9	–4.2 (–16.0, 2.1)	**0.80**
5	–6.3 (–11.0, 4.7)	0.15	–6.7 (–10.6, 3.62)	0.058	–6.4. (–12.0, 4.4)	**0.13**
≥6	–8.8 (–12.0, –1.5)	**0.004**	–9.7 (–12.8, –1.7)	**<0.001**	–9.2 (–13.0, –2.0)	**0.002**
**Trend test**	**<0.001**		**<0.001**		**<0.001**	

CI, Confidence interval; DI-GM, dietary index for gut microbiota. The DI-GM ranges from 0–14 and grouped according to 0–3, 4, 5, and ≥ 6. Model I was unadjusted for covariates; Model II was adjusted for age, sex, race, educational level and poverty–income ratio; Model III was adjusted for age, sex, race, educational level and poverty–income ratio, BMI (body mass index), smoking status, alcohol consumption, hypertension and diabetes. Bold values indicate primary indicators and statistically significant results.

### 3.5 Subgroup analysis and sensitivity analysis

**FIGURE 3:**
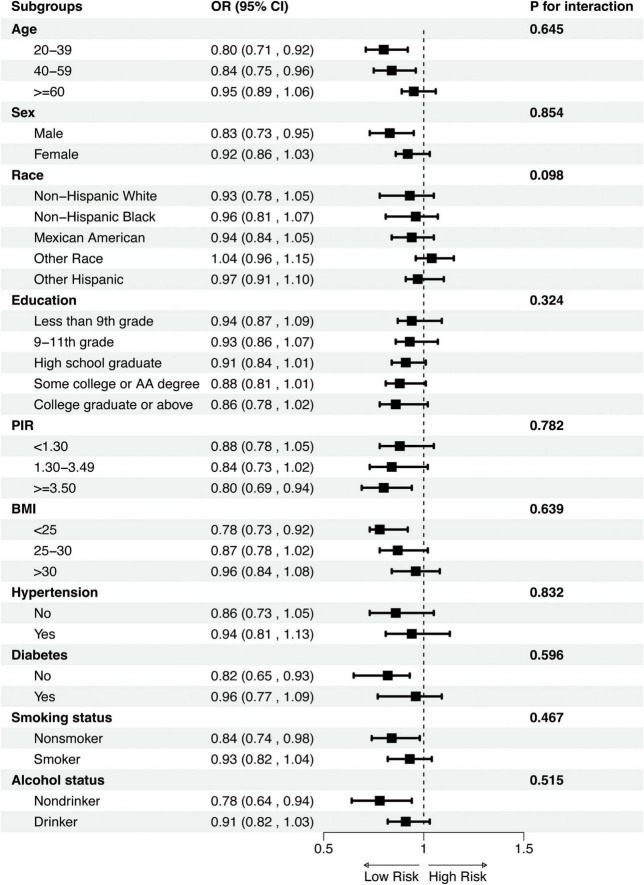
Subgroup analysis of the association between DI_GM and OA. Subgroup analyses were stratified by Age, Sex, Race, Educational, PIR, BMI, Diabetes, Hypertension, Smoking and alcohol status. PIR, poverty-income ratio; BMI, body mass index; OR, Odds Ratio; CI, Confidence Interval.

To evaluate whether the association between DI-GM and OA varies across different subgroups, we performed subgroup analyses and interaction tests (Supplementary Table 2). As shown in Figure 3, no significant interactions were found across subgroups categorized by age (20–39, 40–59, and ≥60 years), sex (male and female), race/ethnicity (non-Hispanic White, non-Hispanic Black, Mexican American, other races, and other Hispanic), educational level (less than grade 9, grades 9–11, high school graduate, some college and college graduate), poverty-income ratio (PIR) (<1.30, 1.30–3.49, and ≥ 3.50), body mass index (BMI) (<25, 25–30, and > 30), hypertension (present and absent), diabetes (present and absent), smoking status (smoker and non-smoker), and alcohol use (drinker and non-drinker). These results suggest that none of these factors significantly influenced the association between DI-GM and OA (all *p* for interaction > 0.05).

PSM analysis results were in agreement with main analysis, further confirmed the stability of the association between DI-GM and OA risk (for details, see Supplementary Tables 3–5).

### 3.6 Mediation analysis

**FIGURE 4:**
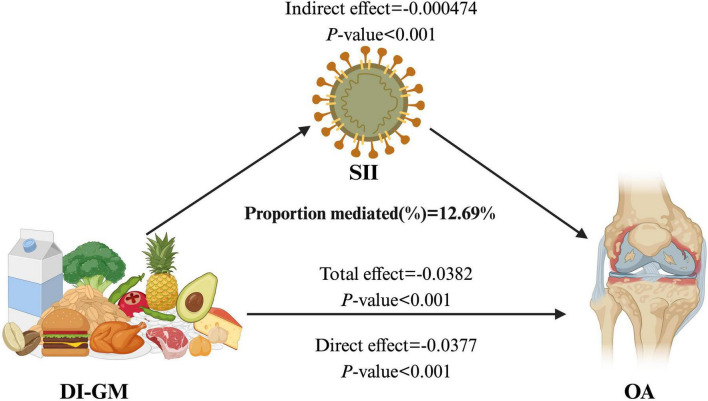
The mediating effect of SII on the relationship between DI-GM and OA. DI-GM, dietary index for gut microbiota; OA, osteoarthritis; SII, systemic inflammation index.

The mediation analysis results indicate that the SII partially mediates the relationship between DI-GM and the prevalence of OA. As illustrated in Figure 4, the total effect of DI-GM on OA is statistically significant (β = –0.0382, *p* < 0.001). The direct effect of DI-GM is β = –0.000474, *p* < 0.001, while the indirect effect through SII is β = –0.000474, *p* < 0.001. Notably, the proportion of the DI-GM-related effect on OA mediated by SII is 12.69% (Supplementary Table 6).

## 4 Discussion

This study explores the relationship between DI-GM and OA risk, and analyzed the mediating effect of SII on this association. Our study results revealed a negative correlation between DI-GM and OA risk, suggesting that higher DI-GM consumption may be associated with a reduced risk of OA. The RCS model showed that the DI-GM and OA were nonlinear relationship. Subgroup analyses indicated that stratification by demographic and clinical characteristics did not significantly affect the association between DI-GM and OA. These findings suggest that the inverse relationship between DI-GM and OA risk remains stable across various populations. Furthermore, the SII was found to partially mediated the relationship between DI-GM and OA, accounting for 12.69% of the total effect.

In recent years, accumulating evidence has indicated a close association between the gut microbiota and OA. Our findings revealed a negative correlation between the DI-GM and the risk of OA, this observation is consistent with previous research, highlighting the protective role of gut microbiota diversity in chronic inflammatory diseases such as OA (27). A diverse gut microbiota, supported by dietary patterns reflected by the DI-GM, is considered essential for maintaining intestinal barrier integrity and regulating systemic inflammation, thereby playing a critical role in mitigating OA pathogenesis (28). Conversely, diminished microbial diversity and dysbiosis have consistently been linked to increased susceptibility to OA (29). Animal studies have demonstrated that a decline in gut microbial diversity in mice compromises intestinal mucosal barrier function and promotes the expansion of pro-inflammatory bacteria, which in turn exacerbating systemic inflammatory, accelerates cartilage degradation, and promotes OA progression (30).

Diet is a critical determinant of gut microbiota composition, with different dietary patterns leading to distinct changes in microbial diversity (31). Our findings support these observations, suggesting that dietary optimization to promote a healthy gut microbiome could reduce the risk of OA. Studies have demonstrated that dietary patterns rich in dietary fiber, probiotics, and anti-inflammatory nutrients positively influence gut microbiota diversity, thus enhancing intestinal barrier integrity and reducing systemic inflammation (32). Similarly, excessive consumption of diets high in saturated fats, refined carbohydrates, and low in dietary fiber promotes the proliferation of pathogenic bacteria, thereby inducing inflammatory responses and metabolic dysregulation (33). A clinical study has indicated that the traditional Mediterranean diet, characterized by abundant intake of fruits, vegetables, whole grains, and fermented foods, could significantly improves microbial diversity and decreases inflammation occurrence (34).

Previous research has demonstrated that gut microbiota plays a crucial role in regulating systemic inflammation and maintaining immune homeostasis (35). In this study, we observed that participants with higher DI-GM scores (≥6) exhibited significantly lower SII. This indicated that diets promoting higher gut microbial diversity may enhance anti-inflammatory capacity, thus potentially reducing OA risk. This finding aligns with prior study indicating that a high level of microbial diversity is typically correlated with enhanced anti-inflammatory capabilities (36). However, at lower DI-GM levels, this association was weaker, suggesting that alternative mechanisms, such as immune modulation, oxidative stress, and antioxidant defenses, may play a role in OA pathogenesis. This effect is potentially attributed to the ability of a diverse gut microbiota to enhance the production of beneficial metabolites, such as the short-chain fatty acids (SCFAs) (37). These metabolites can suppress systemic low-grade inflammation by modulating the T-cells activation (38). Additionally, probiotics and other forms of live microbial foods are well-recognized for promoting beneficial gut flora, which can reduce systemic inflammation and improve metabolic health (39).

In this study, the SII was incorporated into mediation analysis from an exploratory perspective to identify potential mediating pathways linking the DI-GM with OA. This approach aims to provide theoretical support and direction for future longitudinal studies or intervention trials. Specifically, the selection of SII as a mediator was based on the significant differences in gut microbiota composition and diversity observed among OA patients, differences that are potentially associated with aberrant expression of inflammatory markers (13). Consequently, SII serves as an essential mediator, potentially playing a critical role within the “diet–gut microbiota” pathway and its association with OA (40). Our mediation analysis demonstrated that SII significantly mediated the relationship between DI-GM and OA, suggesting that higher DI-GM scores might reduce OA risk by modulating SII levels. A healthy and diverse gut microbiota contributes to maintaining intestinal mucosal barrier integrity, thereby limiting bacterial translocation and systemic exposure to bacterial endotoxins such as lipopolysaccharide (LPS) (41). This, in turn, attenuates systemic inflammatory responses and mitigates chronic inflammation implicated in the pathogenesis of OA.

The strengths of this study are primarily investigating the relationship between the DI-GM, a newly proposed index reflecting gut microbiome diversity, and OA using data from the NHANES database. Additionally, we explored the mediating role of the SII in the association between the DI-GM and OA. Finally, the robustness and reliability of the findings were confirmed through sensitivity analyses, including PSM and subgroup analyses. Despite these strengths of our study, there are notable limitations in our study. Firstly, the cross-sectional design restricts causal inference, making it difficult to determine whether DI-GM directly correlates with a reduced risk of OA. Future studies should be further examined in prospective studies and randomized controlled trials to validate causality. Furthermore, DI-GM was assessed based on 24-h dietary recall data, which may introduce recall bias and affect the accuracy of our findings. Finally, as with many studies, the possibility of residual confounding due to measurement errors in unknown confounders cannot be entirely ruled out.

## 5 Conclusion

In conclusion, our research suggests a negative correlation between the DI-GM and OA in the United States. population. Mediation analyses further explored the mediating role of SII. As a tool for assessing the dietary impact on gut microbiota, the DI-GM may provide valuable insights into new strategies for OA prevention. This underscores the potential of dietary interventions that focus on DI-GM as a strategy for OA management. Dietary guidelines for OA patients should prioritize increasing dietary microbial diversity. In the future research should aim to explore the mechanisms by which DI-GM influences joint health.

## Data Availability

The datasets presented in this study can be found in online repositories. The names of the repository/repositories and accession number(s) can be found below: For a comprehensive description of the NHANES survey methodology and data sources, please visit the website (http://www.cdc.gov/nchs/nhanes/index.htm). All relevant data supporting the findings of this study are included in the article and its Supplementary material.

## Footnotes

1
http://www.R-project.org

## References

[1] Glyn-Jones S Palmer A Agricola R Price A Vincent T Weinans H Osteoarthritis. *Lancet.* (2015) 386:376–87. 10.1016/S0140-6736(14)60802-3 25748615

[2] Katz J Arant K Loeser R. Diagnosis and treatment of hip and knee osteoarthritis: A review. *JAMA.* (2021) 325:568–78. 10.1001/jama.2020.22171 33560326 PMC8225295

[3] Johnson V Hunter D. The epidemiology of osteoarthritis. *Best Pract Res Clin Rheumatol.* (2014) 28:5–15. 10.1016/j.berh.2014.01.004 24792942

[4] Murphy L Helmick C. The impact of osteoarthritis in the united states: A population-health perspective. *Am J Nurs.* (2012) 112:S13–9. 10.1097/01.NAJ.0000412646.80054.21 22373741

[5] Cui A Li H Wang D Zhong J Chen Y Lu H. Global, regional prevalence, incidence and risk factors of knee osteoarthritis in population-based studies. *Eclinicalmedicine.* (2020) 29:100587. 10.1016/j.eclinm.2020.100587 34505846 PMC7704420

[6] Palazzo C Nguyen C Lefèvre-Colau M Rannou F Poiraudeau S. Risk factors and burden of osteoarthritis. *Ann Phys Rehabil Med.* (2016) 59:134–8. 10.1016/j.rehab.2016.01.006 26904959

[7] Flint H Duncan S Scott K Louis P. Links between diet, gut microbiota composition and gut metabolism. *Proc Nutr Soc.* (2015) 74:13–22. 10.1017/S0029665114001463 25268552

[8] Valdes A Walter J Segal E Spector T. Role of the gut microbiota in nutrition and health. *BMJ.* (2018) 361:k2179. 10.1136/bmj.k2179 29899036 PMC6000740

[9] Kase B Liese A Zhang J Murphy E Zhao L Steck S. The development and evaluation of a literature-based dietary index for gut microbiota. *Nutrients.* (2024) 16:1045. 10.3390/nu16071045 38613077 PMC11013161

[10] Ye Z Hu T Wang J Xiao R Liao X Liu M Systemic immune-inflammation index as a potential biomarker of cardiovascular diseases: A systematic review and meta-analysis. *Front Cardiovasc Med.* (2022) 9:933913. 10.3389/fcvm.2022.933913 36003917 PMC9393310

[11] Meng C Liu K. Nonlinear association of the systemic immune-inflammatory index with mortality in diabetic patients. *Front Endocrinol.* (2024) 15:1334811. 10.3389/fendo.2024.1334811 38414824 PMC10898589

[12] He Q Wang Z Mei J Xie C Sun X. Relationship between systemic immune-inflammation index and osteoarthritis: A cross-sectional study from the NHANES 2005–2018. *Front Med.* (2024) 11:1433846. 10.3389/fmed.2024.1433846 39206165 PMC11349521

[13] Zhou Q Liu J Xin L Hu Y Qi Y. Systemic inflammation response index as an emerging biomarker in osteoarthritis patients: A bibliometric and large sample retrospective investigation. *Clin Exp Rheumatol.* (2023) 42:92–103. 10.55563/clinexprheumatol/tygnk6 37497723

[14] Shabbir U Arshad M Sameen A Oh D. Crosstalk between gut and brain in Alzheimer’s Disease: The role of gut microbiota modulation strategies. *Nutrients.* (2021) 13:690. 10.3390/nu13020690 33669988 PMC7924846

[15] Singh A Yau Y Leung K El-Nezami H Lee J. Interaction of polyphenols as antioxidant and anti-inflammatory compounds in brain-liver-gut axis. *Antioxid Basel Switz.* (2020) 9:669. 10.3390/antiox9080669 32722619 PMC7465954

[16] Kabeerdoss J Sandhya P Danda D. Gut inflammation and microbiome in spondyloarthritis. *Rheumatol Int.* (2016) 36:457–68. 10.1007/s00296-015-3414-y 26719306

[17] Takiishi T Fenero C Câmara N. Intestinal barrier and gut microbiota: Shaping our immune responses throughout life. *Tissue Barriers.* (2017) 5:e1373208. 10.1080/21688370.2017.1373208 28956703 PMC5788425

[18] Kalinkovich A Livshits G. A cross talk between dysbiosis and gut-associated immune system governs the development of inflammatory arthropathies. *Semin Arthritis Rheum.* (2019) 49:474–84. 10.1016/J.SEMARTHRIT.2019.05.007 31208713

[19] Zhang X Yang Q Huang J Lin H Luo N Tang H. Association of the newly proposed dietary index for gut microbiota and depression: The mediation effect of phenotypic age and body mass index. *Eur Arch Psychiatry Clin Neurosci.* (2024) [Online ahead of print]. 10.1007/s00406-024-01912-x 39375215

[20] Chen S Sun X Zhou G Jin J Li Z. Association between sensitivity to thyroid hormone indices and the risk of osteoarthritis: An NHANES study. *Eur J Med Res.* (2022) 27:114. 10.1186/s40001-022-00749-1 35820977 PMC9275280

[21] Liu B Wang J Li Y Li K Zhang Q. The association between systemic immune-inflammation index and rheumatoid arthritis: Evidence from NHANES 1999-2018. *Arthritis Res Ther.* (2023) 25:34. 10.1186/s13075-023-03018-6 36871051 PMC9985219

[22] Hu B Yang X Xu Y Sun Y Sun C Guo W Systemic immune-inflammation index predicts prognosis of patients after curative resection for hepatocellular carcinoma. *Clin Cancer Res Off J Am Assoc Cancer Res.* (2014) 20:6212–22. 10.1158/1078-0432.CCR-14-0442 25271081

[23] Xu Q Wang J Li H Gao Y. Association between serum α-klotho levels and osteoarthritis prevalence among middle-aged and older adults: An analysis of the NHANES 2007–2016. *Rev Clínica Esp.* (2024) 224:366–78. 10.1016/j.rceng.2024.04.012 38670226

[24] Gou R Chang X Li Z Pan Y Li G. Association of life’s essential 8 with osteoarthritis in united states adults: Mediating effects of dietary intake of live microbes. *Front Med.* (2023) 10:1297482. 10.3389/fmed.2023.1297482 38179270 PMC10764484

[25] Huang Y Liu X Lin C Chen X Li Y Huang Y Association between the dietary index for gut microbiota and diabetes: The mediating role of phenotypic age and body mass index. *Front Nutr.* (2025) 12:1519346. 10.3389/fnut.2025.1519346 39911810 PMC11794117

[26] Huang G Zhong X Zhang M Xu M Pei B Qian D. The association between lipid biomarkers and osteoarthritis based on the national health and nutrition examination survey and mendelian randomization study. *Sci Rep.* (2024) 14:1357. 10.1038/s41598-024-51523-8 38228737 PMC10792045

[27] Marchese L Contartese D Giavaresi G Di Sarno L Salamanna F. The complex interplay between the gut microbiome and osteoarthritis: A systematic review on potential correlations and therapeutic approaches. *Int J Mol Sci.* (2023) 25:143. 10.3390/ijms25010143 38203314 PMC10778637

[28] Wei Z Li F Pi G. Association between gut microbiota and osteoarthritis: A review of evidence for potential mechanisms and therapeutics. *Front Cell Infect Microbiol.* (2022) 12:812596. 10.3389/fcimb.2022.812596 35372125 PMC8966131

[29] Szychlinska M Di Rosa M Castorina A Mobasheri A Musumeci G. A correlation between intestinal microbiota dysbiosis and osteoarthritis. *Heliyon.* (2019) 5:e01134. 10.1016/j.heliyon.2019.e01134 30671561 PMC6330556

[30] Guan Z Jia J Zhang C Sun T Zhang W Yuan W Gut microbiome dysbiosis alleviates the progression of osteoarthritis in mice. *Clin Sci.* (2020) 134:3159–74. 10.1042/CS20201224 33215637

[31] Beam A Clinger E Hao L. Effect of diet and dietary components on the composition of the gut microbiota. *Nutrients.* (2021) 13:2795. 10.3390/nu13082795 34444955 PMC8398149

[32] Liu T Wang C Wang Y Wang L Ojo O Feng Q The effect of dietary fiber on gut barrier function, gut microbiota, short-chain fatty acids, inflammation and clinical outcomes in critically ill patients: A systematic review and meta-analysis. *JPEN J Parenter Enteral Nutr.* (2021) 46:997–1010. 10.1002/jpen.2319 34951702

[33] Satokari R. High intake of sugar and the balance between pro- and anti-inflammatory gut bacteria. *Nutrients.* (2020) 12:1348. 10.3390/nu12051348 32397233 PMC7284805

[34] Bailey M Holscher H. Microbiome-mediated effects of the Mediterranean diet on inflammation. *Adv Nutr.* (2018) 9:193–206. 10.1093/advances/nmy013 29767701 PMC5952955

[35] Boulangé C Neves A Chilloux J Nicholson J Dumas M. Impact of the gut microbiota on inflammation, obesity, and metabolic disease. *Genome Med.* (2016) 8:42. 10.1186/s13073-016-0303-2 27098727 PMC4839080

[36] Xu J Liang R Zhang W Tian K Li J Chen X *Faecalibacterium prausnitzii*-derived microbial anti-inflammatory molecule regulates intestinal integrity in diabetes mellitus mice via modulating tight junction protein expression. *J Diabetes.* (2020) 12:224–36. 10.1111/1753-0407.12986 31503404 PMC7064962

[37] Ulici V Kelley K Azcarate-Peril M Cleveland R Sartor R Schwartz T Osteoarthritis induced by destabilization of the medial meniscus is reduced in germ-free mice. *Osteoarthritis Cartilage.* (2018) 26:1098–109. 10.1016/j.joca.2018.05.016 29857156 PMC7970023

[38] Levy M Blacher E Elinav E. Microbiome, metabolites and host immunity. *Curr Opin Microbiol.* (2017) 35:8–15. 10.1016/j.mib.2016.10.003 27883933

[39] Dimidi E Cox S Rossi M Whelan K. Fermented foods: Definitions and characteristics, impact on the gut microbiota and effects on gastrointestinal health and disease. *Nutrients.* (2019) 11:1806. 10.3390/nu11081806 31387262 PMC6723656

[40] Randeni N Bordiga M Xu B. A comprehensive review of the triangular relationship among diet–gut microbiota–inflammation. *Int J Mol Sci.* (2024) 25:9366. 10.3390/ijms25179366 39273314 PMC11394685

[41] Shu L Ding Y Xue Q Cai W Deng H. Direct and indirect effects of pathogenic bacteria on the integrity of intestinal barrier. *Ther Adv Gastroenterol.* (2023) 16:17562848231176427. 10.1177/17562848231176427 37274298 PMC10233627

