# Supplementary material for: Association between dietary index for gut microbiota and osteoarthritis in the US population: the mediating role of systemic immune-inflammation index
Source: Front Nutr. 2025 Apr 28;12:1543674. doi: 10.3389/fnut.2025.1543674 (PMC12066459; doi:10.3389/fnut.2025.1543674)
Supplement: Supplementary file 1 [file Table_1.DOCX]

**Supplementary Materials**

**To: Association between dietary index for gut microbiota and osteoarthritis in the US population: a cross-Sectional study from NHANES 2007-2018**

**by: Jiulong Song^1^, Jian Fu^1*^**

**Supplementary Method**

***Weighted analysis***

Weighted analysis is a statistical method that assigns specific weights to different observations within a sample to reflect their varying importance and representativeness within the overall dataset^[1][2]^. This approach effectively corrects biases inherent in sample estimations, thereby enhancing the precision and validity of statistical estimates. In the present study, we employed a weighted analysis approach by incorporating the complex sampling design and mobile examination center (MEC) sample weights recommended by NHANES guidelines, ensuring that our sample accurately represents the extensive diversity and generalizability of the U.S. adult population^[3]^. Consequently, this method significantly enhances the reliability and external validity of our research findings. Moreover, the application of weighted analysis corrects estimation biases arising from sampling errors, thereby further strengthening the accuracy and robustness of our results^[4][5]^.

***Propensity Score Matching***

Propensity Score Matching (PSM) is a widely used statistical method in observational studies, primarily employed to reduce potential confounding bias^[6]^. Propensity score methods generally include four approaches: matching, stratification, weighting, and regression adjustment. The most common technique for calculating propensity scores is logistic regression^[7]^. First, propensity scores are calculated for each study subject using logistic regression based on relevant covariates. Then, matching is performed between the exposure and non-exposure groups according to these calculated scores. Finally, the matched data are analyzed to evaluate the effectiveness of matching and confirm that covariates between groups are well balanced. This allows for a more reliable assessment of the true relationship between exposure factors and study outcomes^[8]^.

**References:**

[1] Meier P. Variance of a weighted mean[J]. Biometrics, 1953, 9: 59.

[2] Doncaster P, Spake R. Correction for bias in meta‐analysis of little‐replicated studies[J]. Methods in Ecology and Evolution, 2017, 9: 634-644.

[3] Beretta L, Tetek J. Better sum estimation via weighted sampling[J]. ACM Transactions on Algorithms, 2021, 20: 1-33.

[4] Cheah J, Roldán J, Ciavolino E, et al. Sampling weight adjustments in partial least squares structural equation modeling: Guidelines and illustrations[J]. Total Quality Management & Business Excellence, 2020, 32: 1594-1613.

[5] Markatou M. Mixture models, robustness, and the weighted likelihood methodology[J]. Biometrics, 2000, 56.

[6] Badhiwala J, Karmur B, Wilson J. Propensity score matching: A powerful tool for analyzing observational nonrandomized data[J]. Clinical Spine Surgery, 2020.

[7] Schober P, Vetter T. Propensity score matching in observational research[J]. Anesthesia and Analgesia, 2020.

[8] Rome L, Patton E. A new heuristic for propensity score matching in observational studies[J]. 2018.

**Supplementary Table**

**Supplementary Table 1. Components and scoring criteria of DI-GM in NHANES.**

**Supplementary Table 2. Subgroup analysis of association between DI-GM and OA.**

**Supplementary Table 3. Baseline characteristics of the study participants grouped by Osteoarthritis status after PSM.**

**Supplementary Table 4. Association between DI-GM and osteoarthritis analyzed using logistic regression after PSM.**

**Supplementary Table 5. The association between DI-GM and systemic immune inflammation index after PSM.**

**Supplementary Table 6. Involvement of SII in mediation the relationship between DI-GM and OA.**

**Supplementary Table 1.** Components and scoring criteria of DI-GM in NHANES.

| Components of DI-GM | Included Foods within the Component | Scoring criteria |
| --- | --- | --- |
| Beneficial to gut microbiota | Fermented dairy (including yogurt, cheese, kefir, sour cream, buttermilk) | For each component beneficial to gut microbiota, a score of 1 if consumption at or above the sex-specific median, else 0  The total score for beneficial components ranged from 0 to 10 |
|  | Chickpeas |  |
|  | Soybean (including Soymilk, Tofu) |  |
|  | Whole grains (grains defined as whole grains, containing the entire grain kernel—the bran, germ, and endosperm) |  |
|  | Dietary fiber |  |
|  | Cranberries |  |
|  | Avocados |  |
|  | Broccoli |  |
|  | Coffee |  |
|  | Green tea |  |
| Unfavorable to gut microbiota | Refined grains (refined grains that do not contain all of the components of the entire grain kernel) | 0 if consumption at or above 40% energy from fat, else 1  For each remaining component unfavorable to gut microbiota, a score of 0 if consumption at or above the sex-specific median, else 1  The total score for unfavourable components ranged from 0 to 4 |
|  | Red meat (including beef, veal, pork, lamb, and game meat; excludes organ meat and cured meat) |  |
|  | Processed meat (including frankfurters, sausages, corned beef, and luncheon meat that are made from beef, pork, or poultry) |  |
|  | High-fat diet (% energy) |  |

DI-GM, dietary index for gut microbiota; NHANES, National Health and Nutrition Examination Survey.

**Supplementary Table 2.** Subgroup analysis of association between DI-GM and OA

|  | **OR (95%CI)** | ***P* for interaction** |
| --- | --- | --- |
| **Age group** |  | **0.645** |
| 20-39 | 0.80 (0.71, 0.92) |  |
| 40-59 | 0.84 (0.75, 0.96) |  |
| ≥60 | 0.87 (0.81, 1.06) |  |
| **Sex** |  | **0.854** |
| Male | 0.83 (0.73, 0.95) |  |
| Female | 0.92 (0.86, 1.03) |  |
| **Race** |  | **0.098** |
| Non-Hispanic White | 0.93 (0.78, 1.05) |  |
| Non-Hispanic Black | 0.96 (0.81, 1.07) |  |
| Mexican American | 0.94 (0.84, 1.05) |  |
| Other Race | 1.04 (0.96, 1.15) |  |
| Other Hispanic | 0.97 (0.91, 1.10) |  |
| **Education level** |  | **0.324** |
| Less than 9th school | 0.94 (0.87, 1.09) |  |
| 9-11th grade | 0.93 (0.86, 1.07) |  |
| High school graduate | 0.91 (0.84, 1.01) |  |
| Some college or AA degree | 0.88 (0.81, 1.01) |  |
| College graduate or above | 0.86 (0.78, 1.02) |  |
| **PIR** |  | **0.782** |
| <1.3 | 0.88 (0.78, 1.05) |  |
| 1.3~3.5 | 0.84 (0.73, 1.02) |  |
| ≥3.5 | 0.80 (0.69, 0.94) |  |
| **BMI** |  | **0.639** |
| <25 | 0.78 (0.73, 0.92) |  |
| 25~30 | 0.87 (0.78, 1.02) |  |
| ≥30 | 0.96 (0.84, 1.08) |  |
| **Hypertension** |  | **0.832** |
| NO | 0.86 (0.73, 1.05) |  |
| Yes | 0.94 (0.81, 1.13) |  |
| **Diabetes** |  | **0.596** |
| NO | 0.82 (0.65, 0.93) |  |
| Yes | 0.96 (0.77, 1.09) |  |
| **Smoking status** |  | **0.467** |
| Nonsmoker | 0.84 (0.74, 0.98) |  |
| Smoker | 0.93 (0.82, 1.04) |  |
| **Alcohol status** |  | **0.515** |
| Nondrinker | 0.78 (0.64, 0.94) |  |
| Drinker | 0.91 (0.82, 1.03) |  |

CI, Confidence interval; DI-GM, dietary index for gut microbiota; NHANES, OR, Odd Ratio; OA, Osteoarthritis; PIR: poverty–income ratio, BMI: body mass index.

**Supplementary Table3** Baseline characteristics of the study participants grouped by Osteoarthritis status after PSM

| **Characteristic** | | **Osteoarthritis** | | |  |
| --- | --- | --- | --- | --- | --- |
|  |  | **Overall,**  **N = 10064** | **Non-OA**  **N = 5032** | **OA**  **N = 5032** | **P-Value** |
| **Age (years), n (%)** |  |  |  |  | <0.001 |
| 20-39 |  | 2013 (24%) | 1420 (32%) | 683 (14.5%) |  |
| 40-59 |  | 3097 (37%) | 1580(36%) | 1517 (38.0%) |  |
| ≥60 |  | 5864 (39%) | 1580 (31%) | 2832 (47.5%) |  |
| **Sex, n (%)** |  |  |  |  | <0.001 |
| Male |  | 4624(44%) | 2431 (46%) | 2193 (42%) |  |
| Female |  | 5440 (51%) | 2601 (54%) | 2839 (58%) |  |
| **Race, n (%)** |  |  |  |  | <0.001 |
| Non-Hispanic White |  | 4897 (73%) | 2270 (70%) | 2627 (76%) |  |
| Non-Hispanic Black |  | 2204 (9.9%) | 1101 (10%) | 1103 (9.7%) |  |
| Mexican American |  | 1171 (6.2%) | 640(7.4%) | 531 (5.0%) |  |
| Other Race |  | 886 (6.2%) | 559 (7.0%) | 327 (5.2%) |  |
| Other Hispanic |  | 906 (4.7%) | 462(5.2%) | 444 (4.1%) |  |
| **Education, n (%)** |  |  |  |  | 0.003 |
| Less than 9th grade |  | 996 (4.9%) | 439 (4.1%) | 557 (5.7%) |  |
| 9-11th grade |  | 1475 (11%) | 714 (10%) | 761 (11%) |  |
| High school graduate |  | 2390 (24%) | 1139 (24%) | 1251 (25%) |  |
| Some college or AA degree |  | 3118 (33%) | 1591 (33%) | 1527 (33%) |  |
| College graduate or above |  | 2085 (27%) | 1149 (33%) | 936 (25%) |  |
| **PIR, n (%)** |  |  |  |  | 0.046 |
| <1.30 |  | 3152 (21%) | 1472 (19%) | 1680 (22%) |  |
| 1.30-3.49 |  | 3945 (37%) | 2009 (37%) | 1936 (36% |  |
| ≥3.50 |  | 2967 (43%) | 1551 (44%) | 1416 (42%) |  |
| **BMI (Kg/m2)** |  |  |  |  | <0.001 |
| <25 |  | 2472 (25%) | 1362 (33%) | 1110 (22%) |  |
| 25-30 |  | 3273 (43%) | 1655 (33%) | 1618 (32%) |  |
| >30 |  | 4319 (43%) | 2015 (34%) | 2034 (46%) |  |
| **Hypertension, n (%)** |  |  |  |  | <0.001 |
| Yes |  | 4736 (42%) | 1997 (35%) | 2739 (50%) |  |
| No |  | 5328 (58%) | 3035(65%) | 2293 (50%) |  |
| **Diabetes, n (%)** |  |  |  |  | <0.001 |
| Yes |  | 1800 (14%) | 725 (11%) | 1075 (17%) |  |
| No |  | 8264 (90%) | 4703(89%) | 3957 (83%) |  |
| **Smoking, n (%)** |  |  |  |  | <0.001 |
| Nonsmoker |  | 4993 (50%) | 2661(53%) | 2332 (47%) |  |
| Smoker |  | 5071 (50%) | 2372 (47%) | 2700 (53%) |  |
| **Alcohol, n (%)** |  |  |  |  | 0.006 |
| Nondrinker |  | 2334 (18%) | 1087(17%) | 1247 (20%) |  |
| Drinker |  | 7730 (82%) | 3945 (83%) | 3785 (80%) |  |
| **SII (mean ± SE)** |  | 557± (3.08) | 545± (3.13) | 570± (3.49) | <0.001 |
| **DI_GM (mean ± SE)** |  | 5.09 ± (0.02) | 5.12 ± (0.06) | 4.80 ± (0.04) | <0.001 |
| **DI_GM n (%)** |  |  |  |  | <0.001 |
| 0-3 |  | 2349 (22%) | 846 (17%) | 1503 (27%) |  |
| 4 |  | 2230 (21%) | 1258 (24%) | 972 (19%) |  |
| 5 |  | 2210 (21%) | 1229 (23%) | 981 (19%) |  |
| ≥6 |  | 3275 (36%) | 1699 (36%) | 1576 (35%) |  |

OA: Osteoarthritis, PIR: poverty–income ratio, BMI: body mass index, SII: systemic inflammation index, SE: standard errors, DI-GM, dietary index for gut microbiota. The DI-GM ranges from 0–14 and grouped according to 0–3, 4, 5, and ≥ 6. PSM, Propensity Score Matching.

**Supplementary Table 4.** Association between DI-GM and osteoarthritis analyzed using logistic regression after PSM

|  | **Model I** |  | **Model II** |  | **Model III** |
| --- | --- | --- | --- | --- | --- |
|  | **OR (95% CI) *P* value** |  | **OR (95% CI) *P* value** |  | **OR (95% CI) *P* value** |
| **DI-GM** | 0.94 (0.90,0.98) **0.001** |  | 0.90 (0.87,0.94) **<0.001** |  | 0.91 (0.87, 0.95) **<0.001** |
| **DI-GM group** | | | | | |
| 0-3  4  5  ≥6 | ref -  0.71 (0.60, 0.85) **<0.001**  0.85 (0.59, 1.01) **0.066**  0.88 (0.75, 1.04) **0.14** |  | ref -  0.65 (0.54, 0.79) **<0.001**  0.78 (0.65, 0.93) **0.008**  0.77 (0.65, 0.93) **0.006** |  | ref -  0.65 (0.53, 0.79) **<0.001**  0.79 (0.66, 0.95) **0.013**  0.79 (0.66,0.95) **0.015** |
| ***P* for trend 0.005**  **<0.001** **<0.001** | | | | | |

PSM, Propensity Score Matching; OR, Odds Ratio; CI, Confidence Interval; DI-GM, dietary index for gut microbiota. The DI-GM ranges from 0–14 and grouped according to 0–3, 4, 5, and ≥ 6. Model I was unadjusted for covariates; Model II was adjusted for age, sex, race, educational level and poverty–income ratio ; Model III was adjusted for age, sex, race, educational level and poverty–income ratio, BMI(body mass index), smoking status, alcohol consumption, hypertension, diabetes, and systemic inflammation index (SII).

**Supplementary Table 5.** The association between DI-GM and systemic immune inflammation index after PSM.

|  | **Model I** |  | **Model II** |  | **Model III** |
| --- | --- | --- | --- | --- | --- |
|  | **β (95% CI) *P* value** |  | **β (95% CI) *P* value** |  | **β (95% CI) *P* value** |
| **DI-GM** | -5.3 (-10.0, -0.58) **0.028** |  | -7.5 (-13.0, -2.1) **0.007** |  | -6.8 (-12.0, -1.4) **0.014** |
| **DI-GM group** | | | | | |
| 0-3  4  5  ≥6 | ref -  -2.5 (-4.3, -0.13) 0.80  -3.8 (-6.2, 1.27) 0.14  -4.0 (-6.7, -1.5) **0.038** |  | ref -  -4.2 (-6.9, 1.31) 0.9  -4.7 (-8.35, 2.48) 0.084  -5.3 (-9.8, -0.38) **0.014** |  | ref -  -3.7 (-6.9, 1.41)  **0.90**  -4.4. (-9.2, 2.47) **0.12**  -5.2 (-10.8, -1.36) **0.024** |
| **Trend test 0.026**  **0.017** **0.021** | | | | | |

PSM, Propensity Score Matching; CI, Confidence Interval; DI-GM, dietary index for gut microbiota; The DI-GM ranges from 0–14 and grouped according to 0–3, 4, 5, and ≥ 6. Model I was unadjusted for covariates; Model II was adjusted for age, sex, race, educational level and poverty–income ratio; Model III was adjusted for age, sex, race, educational level and poverty–income ratio, BMI (body mass index), smoking status, alcohol consumption, hypertension and diabetes

**Supplementary Table 6.** Involvement of SII in mediation the relationship between DI-GM and OA.

|  | Estimate(β) | 95% CI | P-Value |
| --- | --- | --- | --- |
| Total effect  Indirect effect  Direct effect  Proportion mediated | -0.0382  -0.000474  -0.0377  0.01269 | -0.0434, -0.03  -0.000783, 0.001  -0.0430, -0.03  0.0048, 0.02 | <0.001  <0.001  <0.001  <0.001 |

DI-GM, dietary index for gut microbiota; SII, Systemic Immune-Inflammation Index; OA, Osteoarthritis; CI, Confidence Interval.
